# Challenges and opportunities in pediatric residency: an analysis of the increasing number of residents in Italy

**DOI:** 10.1186/s13052-024-01778-8

**Published:** 2024-10-08

**Authors:** Antonio Corsello, Silvia Rotulo, Andrea Santangelo, Alfredo Diana, Federico Rossi, Maria Antonietta Catania, Claudia Aracu, Giuseppe Tiralongo, Francesco Pegoraro

**Affiliations:** 1 National Association of Pediatric Residents (ONSP), Milan, Italy; 2 University of Milan, Milan, Italy; 3 Department of Maternal and Child Health, Umberto I Hospital, Sapienza University of Rome, Rome, Italy; 4 Department of Neurosciences, Rehabilitation, Ophthalmology, Genetics, Maternal and Child Health, University of Genoa, Genoa, Italy; 5 Pediatric Neurology, Pediatric Department, AOUP Santa Chiara Hospital, Pisa, Italy; 6 Section of Pediatrics, Department of Translational Medical Science, University Federico II, Naples, Italy; 7 Pediatric Unit, Department of Clinical, Surgical, Diagnostic and Pediatric Sciences, University of Pavia, Pavia, Italy; 8 Department of Health Promotion, Mother and Child Care, Internal Medicine and Medical Specialties “Giuseppe D’Alessandro”, University of Palermo, Palermo, Italy; 9 UO Neonatologia e Patologia Neonatale, Università Vita Salute, IRCCS Ospedale San Raffaele, Milan, Italy; 10 Academic Department of Pediatrics, University of Rome Tor Vergata, IRCCS Bambino Gesù Children’s Hospital, Rome, Italy; 11 Department of Experimental and Clinical Medicine, University of Florence, Florence, Italy

**Keywords:** Pediatric residency, Training disparities, Clinical autonomy, Research, Future Healthcare

## Abstract

**Background:**

Pediatric residency in Italy has undergone significant changes in recent years, with a notable increase in the number of pediatric residents. Exploring the implications of this increase, highlighting disparities in training opportunities, and discussing the broader socio-economic impacts on pediatric healthcare, represent a crucial consideration for the healthcare system in the future.

**Main Body:**

The Italian National Association of Pediatric Residents (“Osservatorio Nazionale Specializzandi in Pediatria”, ONSP) conducted an extensive survey among pediatric residents to assess the current state of pediatric residency. Key findings indicate that 50% of respondents believe the number of residents is excessive for the available training opportunities, leading to concerns about the quality of education and hands-on experience. Despite the increased number of residents, the workload has increased, but up to one-third of residents feel that the autonomy provided by their programs is insufficient. Significant disparities in training quality were found across different regions, with notable shortages in neonatology and pediatric emergency departments. Research opportunities are also limited, with only 17% of residents finding the time allocated to research satisfactory, especially in central and southern Italy.

**Conclusion:**

The increase in pediatric residents presents both challenges and opportunities. Addressing these challenges through strategic reforms, such as implementing standardized national curricula, investing in training resources and mobility programs, and enhancing research opportunities, is crucial for the future of pediatric residency in Italy. Ensuring high-quality training for all residents is an ethical and practical obligation that will significantly impact pediatric healthcare.

## Background

The landscape of pediatric residency in Italy has undergone significant changes over recent years, especially after the SARS-CoV-2 pandemic. The number of pediatric residents has increased by 75% between the quinquennial periods of 2014–2018 and 2018–2022, which resulted in a notable shift in the dynamics of pediatric training and practice, both during and after the pandemic [1].

During the pandemic, all residents were significantly involved in various aspects of medical assistance. This close involvement limited educational opportunities as many rotations were restricted or suspended. Pediatric residency programs were particularly affected by these limitations [2]. Furthermore, there was a notable decrease in the number of medical consultations during the first wave [3], along with a reduction in hospitalizations for common pediatric diseases [4, 5]. On the other hand, the pandemic is likely to have caused a significant increase in morbidity and mortality due to the restricted access to pediatric facilities, which inevitably resulted in delayed diagnoses and care [6]. Additionally, emergency department admissions declined during the pandemic in various countries [7, 8], inevitably limiting training opportunities for residents.

Parental fears of potential infections, combined with anti-epidemic measures such as mask-wearing, social distancing, and distance learning, contributed to a reduction in common infectious diseases and subsequent pediatric hospital admissions [9, 10]. Despite these challenges, the pandemic period also created new learning opportunities for medical residents, including the increased accessibility of online lessons, webinars, and masterclasses that could be recorded for future review. However, social distancing measures hindered the typical social interactions crucial for residents’ professional training [11].

Moreover, the COVID-19 pandemic led to an increase in the number of social media accounts used by pediatric residents, which can be seen as a supportive tool for residency training programs, offering insights into residents’ opportunities, lifestyles, and job perspectives during their training [12].

This article aims to explore the implications of these changes through a detailed analysis of a survey conducted among more than 20% of total residents in Italy. It investigates potential disparities in training opportunities and discusses the broader socio-economic impacts on pediatric healthcare. Additionally, the uneven distribution of pediatricians across the national territory poses challenges in delivering consistent and high-quality pediatric care, particularly in rural and underserved communities [13]. Addressing this imbalance is essential to improve healthcare outcomes and ensure that all children, regardless of their location, have access to necessary medical attention [14].

The issue of aging medical professionals is not unique to Italy; it is a global phenomenon affecting many healthcare systems [15, 16]. In Europe, approximately 30% of doctors are aged 55 or older, leading to a significant portion of the medical workforce nearing retirement age [17, 18]. This trend poses challenges in ensuring the continuity of healthcare services, particularly in specialties such as pediatrics, where the experience and mentorship of senior doctors are invaluable.

Similarly, in the United States, the aging physician workforce is a growing concern. A recent report indicates that over 40% of active physicians will be 65 or older within the next decade, highlighting an impending wave of retirements that could exacerbate existing physician shortages [19, 20].

To comprehensively understand the current state of pediatric residency in Italy, the Italian National Association of Pediatric Residents (ONSP) conducted an extensive online cross-sectional survey. The survey was distributed among pediatric residents across Italy, with almost 800 respondents representing over 25% of the current pediatric resident population. The survey covered various aspects of residency training, including workload, research opportunities, clinical autonomy, and educational disparities across regions.

## Main text

### The increase in pediatric residents

The number of pediatric residents has significantly increased from 2113 in 2014–2018 to 3699 in 2018–2022 [21–23]. By the end of 2024, pediatric residents will be more than 4200 [24], and many difficulties will need to be addressed in the future to manage these increases, in terms of adequate education and occupation. This surge is partly attributed to the national effort to address the shortage of pediatricians, with the total number of all residency slots increasing by 92% in less than a decade.

#### Survey findings

The survey was completed by 769 residents, coming from all university hospitals in Italy, and equally distributed among Northern, Central and Southern Italy. More than half of the responders believe the actual number of residents is excessive relative to the available training opportunities and university hospitals, leading to concerns about the quality of education and hands-on experience. Despite the increased number of residents, over 50% report working over the allowed 38 h a week, with 65% working more than 8 h daily.

Clinical autonomy remains a critical issue: one-third of residents feel that the autonomy provided by their programs is insufficient. This lack of autonomy is perceived as a significant barrier to developing necessary clinical skills and confidence​​. The survey revealed notable disparities in training quality across different regions. During their 5-year residency educational training, 52% of residents report they spend less than three months in neonatology (nursery, neonatal intensive care units, delivery room), and 42% spend less than three months in pediatric emergency departments (Table 1). This is a major concern, raised by possible new national legislation allowing pediatric residents to be enrolled as full-time pediatricians within facilities of the Italian National Health System since their first years of residency. On one hand, this norm could result in excellent working opportunities for residents, who are offered full-time stable positions at the end of their residency program; however, on the other hand, if they are not granted adequate autonomy since the beginning of their training program, residents could even face dangerous situations when first confronted with a critical clinical decision, resulting in an overall decrease in the quality of care. Such criticalities are exacerbated concerning highly specialized areas, such as neonatology, one of the most demanding in terms of personnel need and the need for high training required to acquire theoretical and manual skills.

**Table 1:** Results from the survey on the average time spent in different services

	1–2 months	3–4 months	5–7 months	8–9 months	> 9 months
How many months will you attend a pediatric department this year?	22.8%	41%	21%	6.6%	8.7%
During the 5 years, how many months will you spend in an emergency department?	37%	39.3%	8.5%	9.6%	6%
During the 5 years, how many months will you spend in neonatology?	7.6%	44.1%	24.2%	23.7%	0.4%
During the 5 years, how many months will you spend in a secondary care hospital?	27%	30.8%	10.7%	19.1%	12.3%

**Fig. 1:**
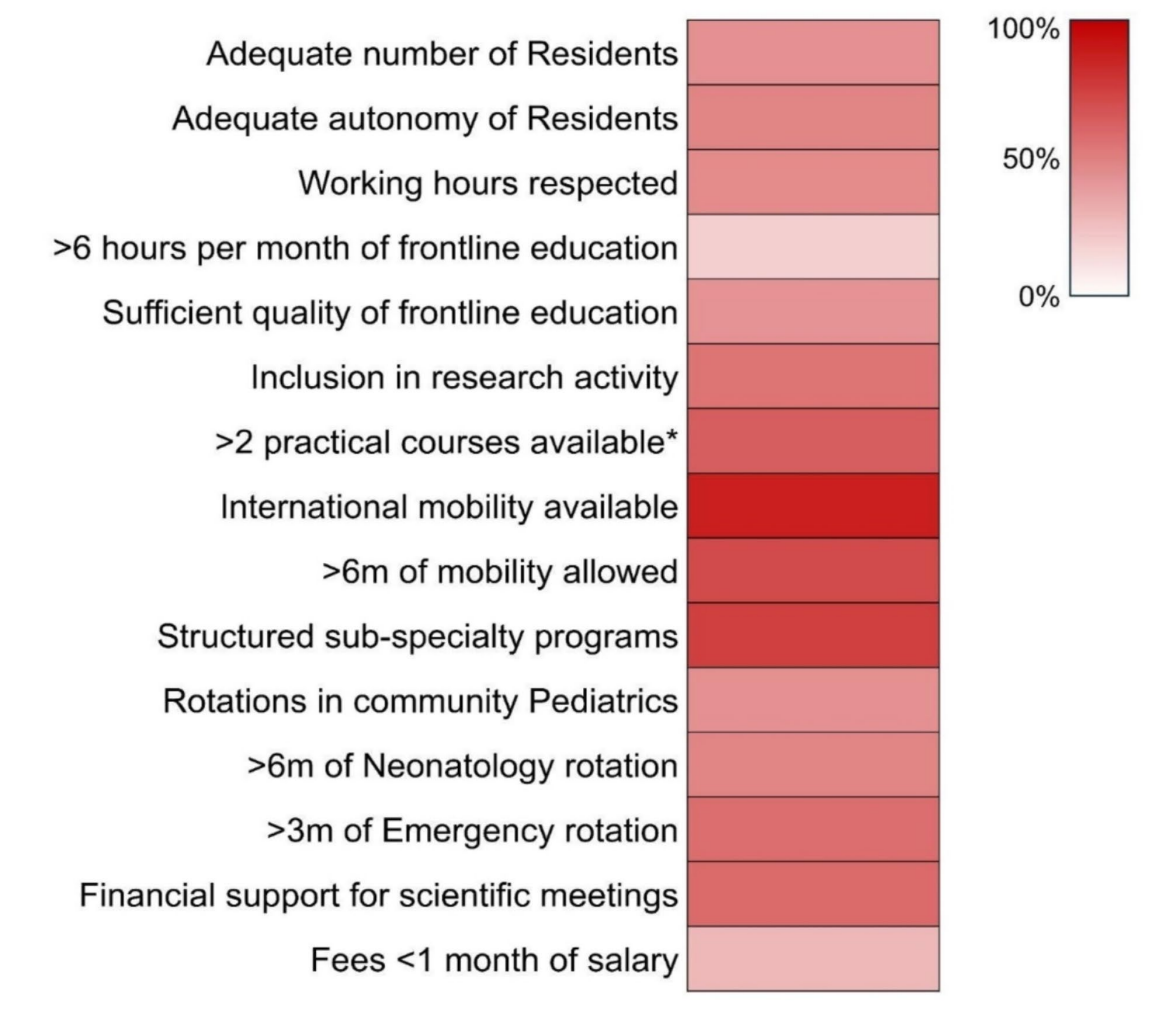
Heat map of the main survey results. *including basic life support, advanced life support, neonatal resuscitation, echography, spirometry, sutures, and others

Research opportunities for pediatric residents are limited, with only 17% finding the time allocated to research satisfactory. This issue is more pronounced in Italy’s central and southern regions, where research infrastructure and support are less developed​​. The main issues emerging from the survey’s answers are summarized in Fig. 1, which shows how major concerns regard frontline educational activities and high fees required by host institutions, which rise in most cases to up to two months of salary. While mobility and sub-specialty programs are usually well developed, there is insufficient coverage of mandatory rotations such as community care, neonatology, and emergency. Moreover, research opportunities are not equally available, despite significant efforts having been made in recent years, also thanks to the ONSP.

#### Challenges and opportunities

The increase in the number of pediatric residents presents both challenges and opportunities. While the higher count of residents is essential to help address the pediatrician shortage, it also strains the existing training infrastructure, leading to reduced quality of education and clinical experience. To guarantee a significant improvement in the pediatric healthcare system, this increase must be accompanied by structural advancements in the Residency programs both in terms of facilities and educational offers.

The aging of the medical workforce and associated retirements are critical issues that need addressing. In Italy, this trend is partly a response to the anticipated retirements in the coming years [25]. The Italian National Healthcare System must proactively address these impending shortages by ensuring that the training and integration of new residents are aligned with future healthcare needs.

High workload and insufficient clinical autonomy are significant concerns. Programs must balance the increased number of residents with adequate hands-on training and supervision. This requires investment in training facilities and faculties to ensure that each resident receives the necessary guidance and opportunities to develop their skills​​​​.

#### Addressing *educational disparities*

Our findings underscore notable disparities in the availability of specialized training opportunities across various fields of pediatric medicine. Specifically, specialties such as pediatric dermatology, cystic fibrosis, nutrition, and Pediatric Intensive Care Units (PICUs) are notably scarce in most residency programs. These deficiencies can be attributed to distinct factors: subspecialties such as dermatology may be less prevalent in hospitals due to the prioritization of more clinically prominent areas; the existence of cystic fibrosis-dedicated centralized centers could justify the challenging training in this field, while the shortage of PICUs may stem from structural inadequacies within hospitals. Following European standards, there should be one PICU bed for every 20,000 to 30,000 children [26]. In Italy, however, the current ratio regrettably stands at 1 bed per 35,856 children, not meeting community standards [27]. Sixteen regions in Italy have less than 25% of the PICU beds recommended by European guidelines [28], indicating substantial deficiencies in critical care infrastructure for pediatric patients nationwide, as well as in the training opportunities provided to pediatricians. Conversely, our data reveal that endocrinology, allergology, and neonatology departments are relatively more available to pediatric residents, and equally distributed in the different regions.

A standalone consideration concerns palliative and end-of-life care. Recently, multiple initiatives, mostly promoted by associations and non-profit organizations, have provided opportunities to residents in Pediatrics, trying to fill the educational gap in this area and a specific course has been made available and mandatory during the 5-year residency period. However, these initiatives remain preliminary and will require significant implementation to provide on-field training, which is currently unfeasible due to the insufficient availability of structures for pediatric palliative care and pediatric hospices on the national territory.

Further considerations could include the possibility of offering residents interdisciplinary rotations in fields such as radiology and dentistry. As suggested by other authors, radiologists could enhance pediatric residents’ understanding of the appropriate use of imaging, while dentists could improve pediatricians’ knowledge of preventive care and children’s oral health through targeted training programs [29, 30].

To address the disparities in training quality, there is a need for a standardized national curriculum that ensures uniform training standards across all regions. This would mitigate the current discrepancies and ensure that most residents receive comprehensive training irrespective of their geographic location. Improving research opportunities is crucial for the professional development of pediatric residents. Establishing dedicated research time and resources can foster a culture of scientific inquiry and innovation among future pediatricians, particularly in regions with currently limited research infrastructure​​.

#### *Socio-*economic *and ethical considerations*

The rise in pediatric residents has significant socio-economic impacts. On one hand, it promises to alleviate the pediatrician shortage, especially in underserved areas. On the other hand, the strain on training resources could lead to a decline in the overall quality of pediatric care if not addressed adequately​​. The ethical implications of these changes must also be considered. Ensuring that all residents receive high-quality training is not only a matter of educational policy but also an ethical obligation to future patients who will rely on these trained professionals for their healthcare needs​​​​.

The progressive level of autonomy for medical trainees is a key factor in their professional development. Currently, trainees enjoy a level of autonomy and opportunities comparable to their more experienced colleagues, which is essential for building confidence and competence in their medical practice. However, there is an urgent need for reform to enhance the dignity and recognition of these residents based on their level of autonomy. According to our data, following the SARS-CoV-2 pandemic, nearly all Italian Pediatric schools (93%) have resumed in-person or hybrid learning (a combination of face-to-face classes and webinars). However, this comes at an average annual cost of 1500–2500 euros in tuition fees, more than a month’s salary from an Italian resident’s salary. Moreover, in comparison to other European countries where first-year residents receive similar salaries, pediatric residents in these countries are paid an incremental basic salary starting at approximately 1750 euros per month, which increases to over 3000 euros per month by the end of their training [31]. These considerations prompt us to reflect on the current state of the Italian education and healthcare system. Even if not yet being classified as “specialists,” residents should still be considered “professionals.” Despite their crucial role, compared to many other high-income countries, Italian residents are underpaid and face uncertainty about their future career prospects, particularly in terms of research and subspecialties. The lack of adequate compensation and job security may worsen burnout syndrome and can deter talented residents from pursuing and improving their competencies and abandoning high-formation centers [32]. Moreover, night shifts are among the most challenging aspects of residency. Extended sleepless hours, high patient expectations, on-call duties, overcrowded emergency departments, intensive neonatal care, unstable patients, and unclear treatment protocols significantly contribute to the risk of burnout [31]. A comprehensive reform is necessary to ensure fair wages, better working conditions, and clear career pathways, thereby providing these dedicated professionals with the stability and respect they deserve.

#### *Further* considerations *on the Italian national healthcare system*

A decade ago, fewer than one in ten doctors in Italy were residents. As of the end of 2023, with the entry of new colleagues and the expected growing number of hospital and community retirements [33], one in five doctors in Italy is a resident. This led to overcrowded university departments and tertiary hospitals, with residents still legally classified as students rather than workers. These numbers should compel the politicians to engage in a collective reflection on the necessity of ensuring adequate training over the next decade. This includes addressing the many contradictions and obstacles that will come to the surface in the next future if the medical community does not effectively engage with institutions, particularly the Ministries of University and Health.

Therefore, it is not surprising that many colleagues, rather than investing additional years in self-funded study amidst the real demand, prefer more gratifying non-specialist medical activities immediately available post-graduation, where they are recognized as professionals and not just young post-graduates. This is not merely a matter of costs but also professional dignity. The current conditions for young doctors in Italy act as a deterrent to investing in their education, resulting in more burdens than rewards in terms of training and quality of life. The concrete risk is a resurgence of the phenomenon observed over the last 20 years [34]. Talented researchers and professionals leave for abroad, as seen in other fields like economics, basic sciences, or engineering. Indeed, the process of reorganizing the residency programs in Europe is likely to be uneasy and complicated by the broad diversity of legislation between countries. Differences in pediatric residency programs across Europe could be transformed by thoughtful planning and cooperation [31].

Table 2 reports possible future directions to improve the next generations of pediatricians, reducing disparities and guaranteeing high-quality educational standards.

**Table 2:** Proposals for future directions

Standardizing training programs	Implementing a national curriculum that ensures consistent training quality across all regions, guaranteeing national/international exchanges for a minimum period of 6 months to all residents.
**Enhancing clinical autonomy**	Providing residents with more opportunities for independent clinical practice under supervision since the first years.
**Progressive autonomy**	Ensure a legalized progressive level of autonomy for residents, allowing them to take on greater responsibility in diagnosis and therapy starting from their third year, especially in light of recent changes in early assumption in primary/secondary hospitals.
**Increasing training resources**	Investing in training facilities and faculties to accommodate the increased number of residents.
**Uniform subspecialty training opportunities**	Guarantee uniform opportunities for all residents to train in subspecialties during the final two years, particularly focusing on community medicine and emergency medicine.
**Promoting research**	Allocating dedicated research time and resources to encourage scientific inquiry.

The challenge for the Italian healthcare system will be to seize the opportunity presented by the much-desired increase in subspecialized and well-trained doctors, a positive outcome of the recent pandemic. The goal should be to make the post-graduate training network a uniform and high-quality experience across the entire national territory, addressing many contradictions and, most importantly, preventing this increase from becoming an obstacle to comprehensive and adequate training.

## Conclusions

The rapid increase in pediatric residents in Italy represents a critical step towards addressing the pediatrician shortage and adapting to the evolving demands of healthcare. However, this is just the start of the process, which needs to be followed by radical structural measures to ensure better education for the future new specialists in Pediatrics. Indeed, this abrupt increase in resident numbers brings to light several significant challenges that must be addressed to ensure the quality of training and patient care, balancing the increase in residents and managing the impending retirements of senior pediatricians, to secure the future of healthcare delivery for the next generation. By implementing standardized training programs, enhancing research opportunities, and addressing regional disparities, Italy can ensure that its future pediatricians are well-equipped to meet the healthcare needs of children across the country. Research opportunities and dedicated research time for pediatric residents are currently limited, with only a small percentage finding the allocated time for research satisfactory​​, particularly in the central and southern regions. The future of pediatric residency in Italy hinges on addressing these challenges through strategic reforms.

## Data Availability

The datasets generated during and/or analyzed during the current study are available from the corresponding author upon reasonable request.

## Abbreviations

ONSP: National Association of Pediatric Residents
PICU: Pediatric Intensive Care Unit
